# Complete Genome Sequence of a Cylindrospermopsin-Producing Cyanobacterium, *Cylindrospermopsis raciborskii* CS505, Containing a Circular Chromosome and a Single Extrachromosomal Element

**DOI:** 10.1128/genomeA.00823-16

**Published:** 2016-08-25

**Authors:** Juan J. Fuentes-Valdés, Alvaro M. Plominsky, Eric E. Allen, Javier Tamames, Mónica Vásquez

**Affiliations:** a Department of Chemical and Bioprocess Engineering, School of Engineering, Pontificia Universidad Católica de Chile, Santiago, Chile; b Department of Molecular Genetics and Microbiology, Faculty of Biological Sciences, Pontificia Universidad Católica de Chile, Santiago, Chile; c Department of Oceanography, Universidad de Concepción, Concepción, Chile; d Instituto Milenio de Oceanografía, Universidad de Concepción, Concepción, Chile; e Marine Biology Research Division, Scripps Institution of Oceanography, University of California San Diego, La Jolla, California, USA; f Division of Biological Sciences, University of California San Diego, La Jolla, California, USA; g Centro Nacional de Biotecnología (CNB-CSIC), Madrid, Spain

## Abstract

*Cylindrospermopsis raciborskii* is a freshwater cyanobacterium producing bloom events and toxicity in drinking water source reservoirs. We present the first genome sequence for *C. raciborskii* CS505 (Australia), containing one 4.1-Mbp chromosome and one 110-Kbp plasmid having G+C contents of 40.3% (3933 genes) and 39.3% (111 genes), respectively.

## GENOME ANNOUNCEMENT

*Cylindrospermopsis raciborskii* is defined as a planktonic nitrogen-fixing freshwater cyanobacterium (1). Strain CS505 (CSIRO culture collection) was isolated from Solomon Dam, North Queensland, Australia (2) and characterized based on its production of the hepatotoxin cylindrospermopsin (CYL), a potent protein synthesis inhibitor (3). In 2010, the draft genome of this strain was analyzed by a combination of 454 and Sanger sequencing, yielding 95 scaffolds with a total length of 3,879,017 bp (4). The cluster associated with the synthesis of CYL toxin was identified, as it had been previously described (5). The strain was grown in MLA liquid medium (6) at 25°C to 28°C under 12:12-h light/dark with a photon flux density of 40 µmol·m^−2^·s^−1^ with no aeration.

In this work, the full genome of the *C. raciborskii* CS505 strain was sequenced using the Pacific Biosciences (PacBio) RS II single-molecule real-time (SMRT) whole-genome sequencing system and assembled using the hierarchical genome assembly process (HGAP) implemented in the PacBio SMRT Analysis software suite (version 2.2.0). The assembly resulted in six scaffolds with a total length of 4,159,260 bp. The average length of the scaffolds was 693,210 bp, with longest and shortest scaffolds sizes of 4,011,384 bp and 2,519 bp, respectively. CheckM analysis (7) indicated a genome completeness of 99.85% with a contamination level of 0.22% and no strain heterogeneity identified. The complete genome and plasmid were annotated using RAST (8) and curated using GenomeMatcher (9).

### Accession number(s).

This whole-genome shotgun project has been deposited at DDBJ/ENA/GenBank under the accession number LYXA00000000. The version described in this paper is version LYXA01000000.
